# Racial disparities in TAVR outcomes in patients with cancer

**DOI:** 10.3389/fcvm.2024.1416092

**Published:** 2024-09-11

**Authors:** Ethan D. Kotloff, Yash Desai, Rohan Desai, Christopher Messner, Sergey Gnilopyat, Mark Sonbol, Abdullah Aljudaibi, Ai Tarui, Juwan Ives, Nisarg Shah, Ishan Vaish, Diljon Chahal, Brian Barr, Manu Mysore

**Affiliations:** ^1^ Department of Medicine, University of Maryland School of Medicine, Baltimore, MD, United States; ^2^ Division of Cardiovascular Medicine, University of Maryland School of Medicine, Baltimore, MD, United States

**Keywords:** cardio-oncology, racial disparities, social determinants of health, aortic stenosis, TAVR

## Abstract

**Background:**

Advances in cancer therapies and improvement in survival of cancer patients have led to a growing number of patients with both cancer and severe aortic stenosis (AS). Transcatheter aortic valve replacement (TAVR) has been shown to be a safe and effective treatment option for this patient population. There are established racial disparities in utilization and outcomes of both cancer treatments and TAVR. However, the effect of race on TAVR outcomes in cancer patients has not been studied.

**Objectives:**

The purpose of this study was to investigate racial disparities in outcomes of TAVR in cancer patients.

**Methods:**

343 patients with cancer who underwent TAVR at a single center over a 6-year period were included in the study. The primary endpoint was a composite of 1-year mortality, stroke, and bleeding. Secondary outcomes included individual components of the primary endpoint as well as 30-day mortality, structural complications, vascular access complications, and conduction system complications. Outcomes were compared between black and white patients by comparing incidence rates.

**Results:**

Baseline characteristics including age, sex, BMI, medical comorbidities, STS score, and echocardiographic parameters were similar between races, aside from significantly higher rates of CKD (50.0% vs. 26.6%, *p* = 0.005) and ESRD (18.4% vs. 4.9%, *p* = 0.005) in black compared to white cancer patients. There was a trend toward worse outcomes in black cancer patients with regard to a composite endpoint of 1-year mortality, stroke, and major bleeding (35.7% vs. 22.6%, *p* = 0.095), primarily driven by higher 1-year mortality (31.0% vs. 17.6%, *p* = 0.065). 30-day mortality was twice as high in black cancer patients than in white cancer patients (4.8% vs. 2.3%, *p* = 0.018).

**Conclusions:**

There is a trend toward worse TAVR outcomes in black cancer patients, with higher periprocedural complication rates and mortality, compared to white cancer patients. Further studies are needed to elucidate the structural, socioeconomic, and biological factors that contribute to racial differences in outcomes.

## 1 Introduction

Advances in cancer treatment and the resulting improvement in survival of cancer patients have led to a growing population of older adults with both cancer and AS. One of the treatment options for AS is TAVR, which has seen a rapid rise in utilization over the past decade from tens of thousands to over one hundred thousand cases annually (1). As cancer therapies continue to improve, the prognosis for these patients may be limited more by AS than by cancer. Moreover, treatment of their AS may allow them to receive more aggressive and optimal oncologic care. Several studies have evaluated the effect of cancer on TAVR outcomes, with the majority showing similar short-term mortality and periprocedural complication rates in cancer patients compared to controls. Based on the existing data, patients with cancer and concomitant severe AS should be considered for TAVR.

Cancer and cardiovascular disease (CVD) are the leading causes of death in the United States with disproportionate burden of illness among black individuals. Black adults have the highest incidence and mortality of any racial group in the US across multiple cancer types and experience higher burden of CVD risk factors and CVD-related mortality than white adults (2). Within the field of cardio-oncology, limited studies have shown that black cancer patients experience higher incidence of cardiotoxicity and higher rates of adverse cardiovascular outcomes compared to white cancer patients (3). Such disparities are postulated to result from a complex interplay between structural, social, environmental, behavioral, and biological factors, which include but are not limited to differences in access to care, socioeconomic status, exposure to environmental pollutants and psychosocial stressors, and genetic background (4). Studies exploring racial disparities in TAVR outcomes are sparse but have shown comparable rates of short-term mortality and periprocedural complications between black and white patients. The aim of this study, therefore, was to investigate the impact of race on TAVR outcomes in a cohort of patients with cancer.

## 2 Materials and methods

### 2.1 Patients

The charts of all patients who underwent TAVR at the University of Maryland Medical Center from November 2016 through August 2022 were retrospectively screened. Of these, 352 patients were identified as having a remote history of or active cancer, defined as having been diagnosed or receiving cancer-related therapy within 1 year of TAVR. Local skin cancers not requiring systemic therapy were not included. 9 patients who identified as either Asian or Hispanic were excluded from the study, leaving 343 patients who identified as either white or black. Race was determined by patient's self-reporting. Medical records were reviewed and baseline characteristics, cancer type and treatment, and TAVR outcomes were recorded to evaluate for racial disparities. The study was approved by the University of Maryland Baltimore (UMB) Institutional Review Board (IRB).

### 2.2 Endpoint definition

The primary endpoint was a composite of 1-year mortality, stroke, and clinically significant (BARC 2 or greater) bleeding. Secondary outcomes included individual components of the primary outcome as well as 30-day mortality, new left bundle branch block, complete heart block, need for permanent pacemaker implantation, structural complications (i.e., ventricular perforation and cardiac tamponade), vascular access complications (i.e., femoral artery pseudoaneurysm), and acute renal failure.

### 2.3 Statistical analysis

Statistical analysis was conducted using RStudio. Baseline characteristics of the patients were compared by race using an unpaired, two-tailed Student's *t*-test for continuous variables and chi-square test for categorical variables. Statistical significance was established at *p* < 0.05. Composite outcomes were derived by combining the incidence of these outcomes and expressing them as percentages of individuals meeting the specified criteria.

## 3 Results

### 3.1 Baseline characteristics

Baseline characteristics of the patients by race are presented in Table 1. Of 343 patients included in the study, 42 (12.2%) identified as black. 49.0% of patients identified as female and gender was distributed equally between races. The mean age at the time of TAVR was 78.9 years and was not significantly different between black and white patients. Rates of cardiovascular risk factors and medical comorbidities were largely comparable between races. However, black patients had significantly higher rates of CKD (50.0% vs. 26.6%, *p* = 0.005) and ESRD (18.4% vs. 4.9%, *p* = 0.005) than white patients. Baseline echocardiographic parameters including left ventricular ejection fraction (54.9% vs. 54.0%, *p* = 0.67) and mean pressure gradient across the aortic valve prior to TAVR (40.94 mmHg vs. 40.14 mmHg, *p* = 0.75) were comparable between black and white patients.

**Table 1:** Baseline characteristics by race.

	Black *n* = 42 (12.2)	White *n* = 301 (87.8)	*P*-value
Age at TAVR (years)	77.8 ± 10.7	79.1 ± 8.4	0.37
Male	22 (52.4)	153 (50.8)	0.98
BMI (kg/m^2^)	30.5 ± 7.9	29.6 ± 9.6	0.57
Tobacco use	21 (50.0)	159 (52.8)	0.86
Comorbid conditions			
Hypertension	37 (88.1)	261 (88.8)	1.00
Hyperlipidemia	31 (79.5)	233 (80.6)	1.00
Diabetes	20 (48.8)	111 (40.1)	0.38
Coronary artery disease	23 (57.5)	158 (57.7)	1.00
Atrial fibrillation	15 (38.5)	119 (43.1)	0.71
End stage renal disease	7 (18.4)	13 (4.9)	0.005*
Echocardiographic parameters			
Left ventricular ejection fraction (%)	54.9 ± 14.2	54.0 ± 12.3	0.67
AV mean PG pre-TAVR (mmHg)	40.94 ± 12.48	40.14 ± 15.78	0.75

Values are mean ± SD or *n* (%).

* Signifies statistical significance.

Characterization of cancer types and cancer therapies are provided in Tables 2, 3. The most prevalent malignancies were breast (17.2%), prostate (15.5%), genitourinary (9.9%), hematological (9.6%), and gastrointestinal (9.3%). 24.2% of patients had multiple cancers. 22.2% of patients had active cancer at the time of TAVR. 64% of patients underwent cancer-related surgery, 34.4% received radiotherapy, 23.6% received chemotherapy, 8.2% received targeted or immunotherapy, and 11.1% received hormonal therapy.

**Table 2:** Cancer types by race.

	Black	White
Breast	6 (14.3)	53 (17.6)
CNS	0 (0.0)	1 (0.3)
ENT	0 (0.0)	6 (2.0)
Gastrointestinal	3 (7.1)	29 (9.6)
Genitourinary	5 (11.9)	29 (9.6)
Gynecological	2 (4.8)	15 (5.0)
Hematological	2 (4.8)	31 (10.3)
Pulmonary	1 (2.4)	11 (3.7)
Melanoma	0 (0.0)	2 (0.7)
Multiple	12 (28.6)	71 (23.6)
Prostate	10 (23.8)	43 (14.3)
Sarcoma	0 (0.0)	1 (0.3)
SCC skin	0 (0.0)	1 (0.3)
Thyroid	1 (2.4)	8 (2.7)

**Table 3:** Cancer therapies by race.

	Black	White	Total	*P*-value
Surgery	30 (71.4)	191 (63.5)	221 (64.4)	0.40
Radiotherapy	11 (26.2)	107 (35.5)	118 (34.4)	0.23
Chemotherapy	7 (16.7)	74 (24.6)	81 (23.6)	0.35
Targeted therapy	2 (4.8)	15 (5.0)	17 (5.0)	1.00
Immunotherapy	2 (4.8)	10 (3.3)	12 (3.5)	0.98
Hormonal therapy	6 (14.3)	32 (10.7)	38 (11.1)	0.66

### 3.2 Outcomes

Outcomes by race are provided in Table 4.

**Table 4:** Mortality and periprocedural outcomes by race.

Outcome	Black *n* = 42 (12.2)	White *n* = 301 (87.8)	*P*-value
Primary outcomes			
1-year mortality	13 (31.0)	53 (17.6)	0.065
Stroke	2 (4.8)	7 (2.3)	0.68
Major bleeding	5 (11.9)	20 (6.6)	0.36
Composite endpoint (1-year mortality, stroke, major bleeding)	15 (35.7)	68 (22.6)	0.095
Secondary outcomes			
30-day mortality	2 (4.8)	7 (2.3)	0.018*
Acute renal failure	0 (0)	2 (0.07)	1.0
Structural complication	2 (4.8)	3 (1.0)	0.222
Permanent pacemaker implantation	6 (14.3)	32 (10.6)	0.657
AV mean PG post-TAVR (mmHg)	11.40 ± 8.98	9.08 ± 4.85	0.01*

* Signifies statistical significance.

### 3.3 Composite endpoint

A total of 83 (24.2%) patients met the composite endpoint of 1-year mortality, stroke, or bleeding. A higher percentage of black patients than white patients met the composite endpoint (35.7% vs. 22.6%, *p* = 0.095).

### 3.4 Mortality

30-day mortality was low overall (2.6%), but significantly higher in black than white patients (4.8% vs. 2.3%, *p* = 0.018). 1-year mortality was also higher in black patients compared to white patients but did not meet statistical significance (31.0% vs. 17.6%, *p* = 0.065).

### 3.5 Periprocedural complications

Black patients had higher incidences of new left bundle branch block, complete heart block, permanent pacemaker implantation, structural complications, vascular access complications, stroke, and BARC 2 or greater bleeding. However, none of these differences were statistically significant. No black patients experienced acute renal failure compared to 2 white patients. Mean pressure gradient across the aortic valve was significantly higher in black patients post-TAVR than in white patients (11.40 mmHg vs. 9.08 mmHg, *p* = 0.01).

## 4 Discussion

The purpose of this study was to examine the effect of race on TAVR outcomes in a cohort of patients with cancer and concomitant severe AS. The main findings are: (1) TAVR can be safely performed in cancer patients with mortality and periprocedural complication rates comparable to those observed in the general population, (2) there is a trend towards worse TAVR outcomes in black cancer patients compared to white cancer patients with regard to a composite endpoint of 1-year mortality, stroke, and bleeding, (3) the worse outcomes in black cancer patients compared to white cancer patients appear to be driven primarily by higher mortality.

### 4.1 Effect of cancer on TAVR outcomes

Our study adds to the growing literature demonstrating that TAVR is a safe and effective treatment option for cancer patients with severe AS with short-term morbidity and mortality comparable to that of non-cancer patients. Landes et al. performed a retrospective cohort study using the TOP-AS registry that showed similar 30-day mortality and periprocedural complication rates but higher 1-year mortality among cancer patients compared to non-cancer controls (14.8% vs. 9.4%, *p* < 0.001), with half of the deaths in cancer patients being cancer related (5). Similarly, a study by Lind et al. of 1,088 patients who underwent TAVR including 839 controls, 196 patients with stable cancer, and 53 patients with active cancer demonstrated comparable 30-day survival and periprocedural complication rates but reduced 10-year survival in those with active cancer (HR 1.47, 95% CI 1.16–1.87, *p* = 0.001), which was attributed to cancer progression (6). Bendary et al. performed a meta-analysis of 3 studies with a total of 5,162 patients including 368 with active cancer that showed similar all-cause mortality, safety, and efficacy outcomes at 30-day follow-up but significantly higher all-cause mortality at 1 year in patients with cancer compared to patients without cancer (RR 1.71, 95% CI 1.26–2.33, *p* = 0.0006), driven by patients in advanced cancer stages (7). More recently, Aikawa et al. retrospectively studied 122,573 TAVR cases of which 8,013 were performed in patients with active cancer and reported similar in-hospital mortality (aOR 1.06, 95% CI 0.89–1.27, *p* = 0.52) but higher rates of bleeding and readmission in active cancer patients (8). While the present study did not include non-cancer controls, mortality and periprocedural complication rates can be compared to those reported in the literature. In line with the aforementioned studies, 30-day mortality was similar (2.62% vs. 3.32%) while 1-year mortality was higher (19.24% vs. 15.62%) in our patient population compared to the STS-ACC TVT registry for all commercial TAVR performed in the US from 2011 through 2019. Incidences of stroke, major bleeding, vascular access complications, and permanent pacemaker implantation were comparable to rates reported in observational studies and clinical trials.

### 4.2 Racial disparities in utilization of TAVR and cancer treatments

Racial disparities in TAVR utilization have been demonstrated in various studies with black patients consistently underrepresented in the TAVR population. Based on US census data from 2021, black individuals represent 9% of Americans aged 65 or older. However, in the STS-ACC TVT registry, only 3.98% of patients who underwent TAVR from 2011 to 2019 were black (9). Reasons for the disparity in TAVR use among black individuals relative to their representation in the US population are incompletely understood and likely multifactorial. Studies have suggested that black individuals are at significantly lower risk of developing severe AS than white individuals, despite higher prevalence of traditional risk factors (10). However, a retrospective cohort study by Brennan et al. showed that black patients were significantly less likely than white patients to undergo aortic valve replacement (either SAVR or TAVR) 1 year after diagnosis of symptomatic severe AS, suggesting that there are additional factors separate from disease prevalence that contribute to racial differences in TAVR usage (11). Potential causes include lower socioeconomic status and, in turn, lack of health insurance and access to specialized interventions, lack of trust in the medical system, and lower likelihood of referral for specialized procedures. Indeed, within major metropolitan areas in the US with TAVR programs, zip codes with higher proportions of black patients and lower measures of socioeconomic status (i.e., median household income, Medicaid eligibility) have lower rates of TAVR (12). Furthermore, after diagnosis of severe AS, black patients are more likely to decline TAVR or not be referred to a cardiologist at all (13). In the present study, 12.2% of patients who underwent TAVR were black, almost triple the rate reported in the STS-ACC TVT registry throughout 2019. It is unclear, however, whether this represents an improvement in utilization of TAVR among black patients since 2019 or is simply a result of our distinct study population. Given the high incidence of cancer in black relative to white adults and the urban location of our study site, a higher percentage of black patients is to be expected in our cohort compared with the general TAVR population.

Racial disparities also impact the utilization of cancer treatments. For example, in a cohort of patients with gastrointestinal tract cancer, black patients were 8% less likely to receive chemotherapy and 35% less likely to receive radiotherapy than white patients (14). Similar disparities have been demonstrated in utilization of hormonal therapy for prostate cancer (15). However, in the present study we noted no significant differences in utilization of various cancer treatments between black and white patients. A potential explanation for this could be due to our distinct patient population, which consisted of a higher percentage of black adults compared to the general US population, and therefore, significant emphasis and efforts to provide more equitable care.

### 4.3 Racial disparities in TAVR outcomes

Several studies have explored the impact of race on TAVR outcomes. In an early, single-center retrospective cohort study, Minha et al. found no differences in periprocedural outcomes, 30-day, or 1-year mortality between black and white patients who underwent TAVR (16). Alkhouli et al. performed a retrospective cohort study of 70,221 patients using the STS-ACC TVT registry that also showed no significant difference in the rates of in-hospital or 1-year mortality as well as in-hospital MI, stroke, major bleeding, vascular complications, or new pacemaker requirements between black and white patients. However, black patients had higher rates of rehospitalization for heart failure at 1-year follow-up compared with white patients (HR 1.39, 95% CI 1.16–1.67, *p* < 0.001) for unclear reasons (17). A meta-analysis by Jaiswal et al. that included 3 studies with a total of 102,009 patients similarly demonstrated comparable in-hospital mortality among black and white patients (OR 1.01, 95% CI 0.86–1.19, *p* = 0.93). However, the rates of certain secondary outcomes differed by race: black patients had significantly higher rates of myocardial infarction and acute kidney injury but lower rates of permanent pacemaker implantation (18).

Our study, in contrast, demonstrated a trend towards worse periprocedural outcomes and significantly worse mortality in black compared with white patients. 35.7% of black patients met the composite endpoint of 1-year mortality, stroke, and/or major bleeding compared with 22.6% of white patients (*p* = 0.095), primarily driven by higher 1-year mortality (31.0% vs. 17.6%, *p* = 0.065). Given that cause of death at 1 year is unknown, it is not clear whether the increased 1-year mortality in black patients is primarily due to cancer or cardiovascular death. Crude incidence rates of all periprocedural complications including intraprocedural death, stroke, major bleeding, vascular complications, and conduction system complications were higher in black patients than white patients. However, none of these differences were statistically significant, likely due to the limited sample size. In contrast to prior studies evaluating racial disparities in TAVR outcomes, 30-day mortality was significantly worse in black patients with a mortality rate more than double that of white patients. However, this result should be interpreted with caution given the small number of patients who died within 30 days of TAVR (2 black patients vs. 7 white patients). It is possible that differences in baseline comorbidities, particularly the higher incidence of ESRD in black patients, contributed to differences in outcomes. Indeed, multiple retrospective cohort studies have demonstrated that patients with CKD or ESRD who undergo TAVR have greater short- and long-term mortality and periprocedural complication rates compared with patients without renal dysfunction (19–23). Finally, mean pressure gradient across the aortic valve post-TAVR was significantly higher in black patients compared to white patients, although the difference is unlikely to be clinically significant. Further clinical and translational research is needed to develop a better understanding of the structural, socioeconomic, and biological factors that drive racial differences in TAVR outcomes in cancer patients.

### 4.4 Limitations

This study has several limitations. First, as a retrospective, single-center cohort study, it is susceptible to bias and confounding that is inherent to all observational studies. Second, given the specific nature of our study question, the sample size was limited to 343 total subjects of whom only 42 identified as black. Our study, therefore, was likely not powered to detect true differences in certain secondary outcomes. Moreover, due to limitations in sample size, we could not comment on disparities in outcomes among Asian and Hispanic patients, which warrants further study. Third, cancer-specific data including cancer stage and presence of metastatic disease was not obtained and therefore, cancer-specific prognosis could not be estimated. Fourth, as most deaths occurred outside the hospital and were not recorded in the electronic medical record, mortality data was primarily obtained from obituary records. It is possible that some deaths were missed if obituaries were not readily available online. Furthermore, cause of death was unknown for all patients who died during the follow up period so it is not clear whether patients died primarily from cancer or cardiovascular-related causes. Lastly, socioeconomic and geographic variables, which are likely to contribute to racial disparities in outcomes, were not collected.

## 5 Conclusions

Black patients with cancer and severe AS who undergo TAVR tend to have worse periprocedural outcomes, including significantly higher short-term mortality, in comparison to white patients. Further studies are needed to elucidate the structural, socioeconomic, environmental, behavioral, and biological factors that contribute to racial disparities in outcomes.

## Data Availability

The raw data supporting the conclusions of this article will be made available by the authors, without undue reservation.

## References

[1] Kim KM Arghami A Habib R Daneshmand MA Parsons N Elhalabi Z The society of thoracic surgeons adult cardiac surgery database: 2022 update on outcomes and research. Ann Thorac Surg. (2023) 115(3):566–74. 10.1016/j.athoracsur.2022.12.033 36623634

[2] African American People and Cancer. Available online at: https://www.cdc.gov/cancer/health-equity/groups/african-american.htm (accessed February 3, 2024)

[3] Fazal M Malisa J Rhee JW Witteles RM Rodriguez F. Racial and ethnic disparities in cardio-oncology: a call to action. JACC CardioOncol. (2021) 3(2):201–4. 10.1016/j.jaccao.2021.05.001 34308372 PMC8301207

[4] Zavala VA Bracci PM Carethers JM Carvajal-Carmona L Coggins NB Cruz-Correa MR Cancer health disparities in racial/ethnic minorities in the United States. Br J Cancer. (2021) 124(2):315–32. 10.1038/s41416-020-01038-6 32901135 PMC7852513

[5] Landes U Iakobishvili Z Vronsky D Zusman O Barsheshet A Jaffe R Transcatheter aortic valve replacement in oncology patients with severe aortic stenosis. JACC Cardiovasc Interv. (2019) 12(1):78–86. 10.1016/j.jcin.2018.10.026 30621982

[6] Lind A Totzeck M Mahabadi AA Jánosi RA El Gabry M Ruhparwar A Impact of cancer in patients undergoing transcatheter aortic valve replacement: a single-center study. JACC CardioOncol. (2020) 2(5):735–43. 10.1016/j.jaccao.2020.11.008 34396288 PMC8352296

[7] Bendary A Ramzy A Bendary M Salem M. Transcatheter aortic valve replacement in patients with severe aortic stenosis and active cancer: a systematic review and meta-analysis. Open Heart. (2020) 7(1):e001131. 10.1136/openhrt-2019-001131 32201582 PMC7066604

[8] Aikawa T Kuno T Malik AH Briasoulis A Kolte D Kampaktsis PN Transcatheter aortic valve replacement in patients with or without active cancer. J Am Heart Assoc. (2023) 12(21):e030072. 10.1161/JAHA.123.030072 37889175 PMC10727376

[9] Carroll JD Mack MJ Vemulapalli S Herrmann HC Gleason TG Hanzel G STS-ACC TVT registry of transcatheter aortic valve replacement. Ann Thorac Surg. (2021) 111(2):701–22. 10.1016/j.athoracsur.2020.09.002 33213826

[10] Patel DK Green KD Fudim M Harrell FE Wang TJ Robbins MA. Racial differences in the prevalence of severe aortic stenosis. J Am Heart Assoc. (2014) 3(3):e000879. 10.1161/JAHA.114.000879 24870936 PMC4309086

[11] Matthew Brennan J Leon MB Sheridan P Boero IJ Chen Q Lowenstern A Racial differences in the use of aortic valve replacement for treatment of symptomatic severe aortic valve stenosis in the transcatheter aortic valve replacement era. J Am Heart Assoc. (2020) 9(16):e015879. 10.1161/JAHA.119.015879 32777969 PMC7660794

[12] Nathan AS Yang L Yang N Eberly LA Khatana SAM Dayoub EJ Racial, ethnic, and socioeconomic disparities in access to transcatheter aortic valve replacement within major metropolitan areas. JAMA Cardiol. (2022) 7(2):150–7. 10.1001/jamacardio.2021.4641 34787635 PMC8600460

[13] Sleder A Tackett S Cerasale M Mittal C Isseh I Radjef R Socioeconomic and racial disparities: a case-control study of patients receiving transcatheter aortic valve replacement for severe aortic stenosis. J Racial Ethn Health Disparities. (2017) 4(6):1189–94. 10.1007/s40615-016-0325-x 28039604

[14] Bakkila BF Kerekes D Nunez-Smith M Billingsley KG Ahuja N Wang K Evaluation of racial disparities in quality of care for patients with gastrointestinal tract cancer treated with surgery. JAMA Netw Open. (2022) 5(4):e225664. 10.1001/jamanetworkopen.2022.5664 35377425 PMC8980937

[15] Ma TM Agarwal N Mahal B Barragan-Carrillo R Spratt D Rettig MB Racial and ethnic disparities in use of novel hormonal therapy agents in patients with prostate cancer. JAMA Netw Open. (2023) 6(12):e2345906. 10.1001/jamanetworkopen.2023.45906 38039002 PMC10692845

[16] Minha S Barbash IM Magalhaes MA Ben-Dor I Okubagzi PG Pendyala LK Outcome comparison of African-American and Caucasian patients with severe aortic stenosis subjected to transcatheter aortic valve replacement: a single-center experience. Catheter Cardiovasc Interv. (2015) 85(4):640–7. 10.1002/ccd.25535 24782407

[17] Alkhouli M Holmes DR Jr Carroll JD Li Z Inohara T Kosinski AS Racial disparities in the utilization and outcomes of TAVR: TVT registry report. JACC Cardiovasc Interv. (2019) 12(10):936–48. 10.1016/j.jcin.2019.03.007 31122351

[18] Jaiswal V Peng Ang S Hanif M Savaliya M Vadhera A Raj N The racial disparity among post transcatheter aortic valve replacement outcomes: a meta-analysis. Int J Cardiol Heart Vasc. (2023) 44:101170. 10.1016/j.ijcha.2023.101170 36660201 PMC9843207

[19] Lorente-Ros M Das S Malik A Romeo FJ Aguilar-Gallardo JS Fakhoury M In-hospital outcomes of transcatheter aortic valve replacement in patients with chronic and end-stage renal disease: a nationwide database study. BMC Cardiovasc Disord. (2024) 24(1):21. 10.1186/s12872-023-03684-z 38172786 PMC10765730

[20] Mohananey D Griffin BP Svensson LG Popovic ZB Tuzcu EM Rodriguez LL Comparative outcomes of patients with advanced renal dysfunction undergoing transcatheter aortic valve replacement in the United States from 2011 to 2014. Circ Cardiovasc Interv. (2017) 10(10):e005477. 10.1161/CIRCINTERVENTIONS.117.005477 28951396

[21] Ogami T Kurlansky P Takayama H Ning Y Ali ZA Nazif TM Long-term outcomes of transcatheter aortic valve replacement in patients with end-stage renal disease. J Am Heart Assoc. (2021) 10(16):e019930. 10.1161/JAHA.120.019930 34387093 PMC8475055

[22] Szerlip M Zajarias A Vemalapalli S Brennan M Dai D Maniar H Transcatheter aortic valve replacement in patients with end-stage renal disease. J Am Coll Cardiol. (2019) 73(22):2806–15. 10.1016/j.jacc.2019.03.496 31171086

[23] Gupta T Goel K Kolte D Khera S Villablanca PA Aronow WS Association of chronic kidney disease with in-hospital outcomes of transcatheter aortic valve replacement. JACC Cardiovasc Interv. (2017) 10(20):2050–60. 10.1016/j.jcin.2017.07.044 29050621

